# Little Things

**Published:** 1893-08

**Authors:** W. H. Wright

**Affiliations:** Brandon


### THE DENTAL REGISTER.

VOL. XLVII.] AUGUST, 1893. [No. 8.

### COMMUNICATIONS.

### Little Things.

BY DR. W. H. WRIGHT, OF BRANDON.

Read before the Vermont State Dental Society, March, 1893.

It seems almost useless to write a paper upon the value of little things. It is one of the chief lessons to be learned from the advances which science has made in our day. That it has been achieved by men who were willing to investigate the smallest phenomena, and to expend an amount of care on their experiments which seems foolish only to those who have neither the patience nor the skill to successfully interrogate nature. Macaulay has pointed out the fact that it was the rare combination of the inductive and demonstrative faculties in the mind of Sir Isaac Newton which produced his splendid and revolutionary contributions to science. In him they co-existed in supreme excellence and perfect harmony; the progress of human knowledge has been by a close attention to things long overlooked and whose simplicity concealed their importance. And so, when in the practice of our profession we would assist nature, or successfully imitate her work, we must recognize the importance of proceeding by her methods. A failure to do this has produced disastrous results to many ambitious attempts to extend the boundaries of knowledge.

A few years ago it was claimed that life had been evolved from sterilized infusions and a great step taken in solving the problem of how dead matter became organic. But later and more careful experiments showed that sufficient care had not been exercised to exclude the atmosphere, the horde of infinitesimal germs which supply the fecund principle. The discovery of living matter from non-living is yet to be made. So also it was thought at one time that the original form of matter had been discovered in bathybius, a soft, jelly-like substance, found in bottles lowered to a great depth in the sea. But soon it was proved that the product was the result of chemical action caused by the malt and liquors in the bottles used. Perfectly clean vessels gave no results. But lately the world was delighted to hear of Dr. Koch's discovery of a fluid that would cure consumption; but on trial it has not proved to be the panacea so devoutly to be desired. Some things had been overlooked, small in themselves, yet sufficient to neutralize the efficacy of the compound.

It is evident that he who would enter the realms of nature and explore her secrets must have an eye for little things and be possessed of inexhaustible patience in their observation. And the same is true in applying principles well ascertained to actual cases. In the reparatory process which combats the inroads of disease and seeks to restore parts destroyed there is need of attention to little things. The nearer we can come to nature the better will be the results attained. It can only be by the most careful study and observation that we are warranted in concluding that anything pertaining to our work is relatively unimportant; we must learn to eliminate that which is little, because it has nothing to do with what we seek; and to recognize that which while little is yet vital to success, because without it success cannot be attained. At no point do we need to be more on our guard against being deceived by appearances than in discriminating what is and what is not essential to successful treatment.

Nothing is little which in any way has to do with the production or cure of disease in any part of the human body. The processes of departure from health to disease are in any tissue only to be seen by a skilled observer who has learned to see potent evils in small beginings. The wonderful success of that most eminent of English surgeons, Dr. Dawson Tait, has been due to his carefulness and attention to little things both before and after operating, which had hitherto been deemed of small moment. Illustrations of this principle might be multiplied from every department of theoretical or applied science and will readily be admitted by all who are familiar with the advances in human knowledge. But it is well to remind ourselves even of so obvious a truth, that it may enter as a controlling force in our daily practice. Like all general knowledge, it is of little use until it has been transformed into a habit which is characteristic of all we do. It is well to establish the invaluable habit of investigating a case with thoroughness and getting all the facts in the case before making our inductions. This will be a safeguard against crude opinions formed by overlooking something which is intimately related to the subject in hand. Not infrequently it will be rewarded by a discovery which will render future investigations easy and certain.

It is not needful to remind you that attention to little things is not the only condition of success. The great and the little are not to be confounded or confused. A narrow vision may miss the vital point as well as one too general in its range. A fussiness about details, simply because they are in some way related to our subject may unfit for the expenditure of energy at the point where it will do the most good. But bearing this in mind and exercising ordinary common sense we may apply this principle in our practice with most gratifying results. Even when this concerns only what is accessory to the main work it is still worthy our attention. It should be our principal ambition to do the best possible work in our profession and this cannot be done without attention to details each little in itself, but which together make perfection.

While it is of minor importance it is yet well worth our endeavor to avoid the infliction of all unnecessary pain. This may be a temporary matter soon over; but not so soon forgotten by the sufferer as we would imagine. He may blame us for the pain while he appreciates the good work done. Good work is what he demands and what will redound to our credit, but our patients will recognize the kindness and skill of a deft hand reducing pain to the lowest possible point. It is human to shrink from pain and there is no one who will not submit more readily to treatment by one who is careful in this matter. Ability to do this argues a nice attention to little details, a skilled hand as well as a kind heart, which will amply reward one who cultivates the habit of considering nothing little which adds to the comfort of his patient. The best illustration of my unambitious subject will be found on the beaten path of daily practice rather than in those exceptional cases which by their very rarity put the practitioner on his metal and induce the greatest care.

It is a small thing after the patient is seated in the operating chair, in a highly excited and nervous state, to have an impression taken for an upper plate, that the operator saturate a piece of cotton with cocaine and direct him to place it on the tongue and move it over the roof of the mouth. This will enable him when the impression cup is introduced to bear it without discomfort, so that the cup shall remain in place until the plaster hardens, and will prevent the retching and possible vomiting which sometimes, in patients of sensitive stomachs, render the operation both painful and liable to complete failure. Attention to these minor points often aids the operator as much as it relieves the patient. Again, in opening the vulcanizer if we have used a "Whitney" needless effort may be avoided by a little forethought and our patience and our temper both be improved. If instead of the old methods in which perhaps we have jammed our hands and have felt like breaking a commandment, we have a long-handled, malleable iron wrench, which a village blacksmith can make, for the top of the boiler, and place this in position and lay the vulcanizer on its side on the bench and hit the long handle of the wrench a smart blow or two with a hammer the work is done without friction to hands or feeling.

It will make much difference with our patients, especially the little ones, if we have so kindly a remedy, so wide in the range of its usefulness as phenacetine. This is especially valuable with children in cases of mild febrile reaction of childhood which arises seemingly from slight intestinal irritation, and which is so common during the eruption of the teeth in infancy. It will be found to be most soothing in its influence when administered in doses of from two (2) to four (4) grains according to age, once in three hours. Generally it induces grateful and refreshing sleep and that moist condition so much to be desired. Slight irritation may thus be subdued, which if unrelieved may prove to be quite serious. We should not lose sight of the value of lancing the gums over the approaching tooth to relieve the pressure on the sensitive nerves and thus kindly aid nature in its task of bringing a new tooth into the world of dental usefulness.

I have also found it extremely useful in adult cases where the patient was suffering from acute alveolar abscesses with all the accompanying febrile and distressing symptoms, loss of sleep and general nervous irritability. I have used with great satisfaction to many patients, in doses of from (5) five to (7) seven grains repeated once in (3) three to (4) four hours. The result has been to induce sleep and to reduce the temperature. Phenacetine is certainly one of the most valuable and one of the safest of the products of the coal tar series which are in use to produce hypnotic effects. It is much to have in our hands so good a substitute for quieting pain instead of resorting to the hypodermic injection of morphia, inducing in many cases a habit which eventually wrecks the poor victim. I give some examples of the way this new antipyretic acted with my patients. The wife of a druggist writes me as follows: "I deem it a pleasure to speak a good word for phenacetine as it has proved a very safe and certain drug in relieving severe neuralgic pain as well as allaying high fever and intense nervousness in my little son five years of age, after having two large teeth extracted. He is of a highly excitable temperament and inclined to spasms. The second night after the teeth were out his sleep was troubled with high fever, moanings and twitchings of the muscles. Fearing a fit I carefully aroused him about midnight and followed the directions of our dentist, Dr. W. H. Wright, by giving a four-grain phenacetine powder. The effect was more than satisfactory. In a short time he was in a gentle perspiration and sleeping gently. The succeeding nights gave four-grain powder upon his retiring and thus secured perfect rest without serious after effects. In my own case it was none the less beneficial. After suffering twelve (12) hours with neuralgia of the face, caused in part by a diseased tooth, I took two (5) five-grain tablets, allowing thirty minutes between them. What narcotics and chloroform did not do phenacetine quickly accomplished."

In October last Mrs. S. B. L., who is the daughter of the late celebrated Dr. Horace Greene, of New York, came to me for treatment, suffering intensely from an ulcerated lower bicuspid. Had not slept much for several nights; inflammation extended to the cheek, ear and eyes. As she resided too far away to secure daily local treatment I applied the usual remedies locally and prescribed five (5) grain phenacetine tablets. After taking two she was entirely relieved and had no further trouble. Subsequently the tooth was filled and is now doing good service.

Again, in the latter part of February, I fitted two crowns on to old roots. She went home suffering much from inflammation of the gums. She was obliged to take two tablets for three nights and has had no trouble since.

There is no question in my mind that we have in this new agent a very valuable assistant in fighting the various inflammatory action met with in our practice. But a paper on Little Things should not be allowed to grow to tediousness for that is not a small matter in a world where there are so many sources of worry. Having indicated sufficiently the value of attention to minor matters I must leave to your good judgment its application. It may be a slow but it is a sure way to the best success. The aggregation of these little acts will make the difference between the finished workman and the shiftless practitioner. A great painter was once asked "Why he spent so much time upon the trifling parts of his picture?" He replied, and there is wit as well as wisdom in his words: "Because, perfection is made up of trifles, and perfection is no trifle."

